# An Evidence-Informed Framework to Promote Mental Wellbeing in Elite Sport

**DOI:** 10.3389/fpsyg.2022.780359

**Published:** 2022-02-16

**Authors:** Rosemary Purcell, Vita Pilkington, Serena Carberry, David Reid, Kate Gwyther, Kate Hall, Adam Deacon, Ranjit Manon, Courtney C. Walton, Simon Rice

**Affiliations:** ^1^Orygen, Melbourne, VIC, Australia; ^2^Centre for Youth Mental Health, The University of Melbourne, Melbourne, VIC, Australia; ^3^School of Psychology, University College Dublin, Dublin, Ireland; ^4^Focus Coaching, Melbourne, VIC, Australia; ^5^Australian Football League, Docklands, VIC, Australia; ^6^School of Psychology, Deakin University, Geelong, VIC, Australia; ^7^Consultant Psychiatrist, Melbourne, VIC, Australia; ^8^Department of Psychiatry, Monash University, Clayton, VIC, Australia; ^9^Youth Mood Clinic, Orygen Youth Health, Melbourne, VIC, Australia

**Keywords:** athletes, sport, mental health, wellbeing, psychological, promotion, prevention

## Abstract

Elite athletes, coaches and high-performance staff are exposed to a range of stressors that have been shown to increase their susceptibility to experiencing mental ill-health. Despite this, athletes may be less inclined than the general population to seek support for their mental health due to stigma, perceptions of limited psychological safety within sport to disclose mental health difficulties (e.g., selection concerns) and/or fears of help-seeking signifying weakness in the context of high performance sport. Guidance on the best ways to promote mental health within sporting environments is increasing, though current frameworks and position statements require greater focus on a *whole of system* approach, in which the needs of athlete, coaches and high-performance staff are considered within the context of the broader ecological system in which they operate and perform. This paper synthesizes existing research, reviewed for translatability by mental health professionals working in elite sport, to provide an evidence-informed framework with real world utility to promote mentally healthy environments for all stakeholders in elite sporting organizations, from athletes through to administrators. Recommendations are provided to positively impact the mental wellbeing of athletes and support staff, which may in turn influence athletic performance. This framework is intended to provide sporting organizations with evidence-informed or best practice principles on which they can develop or progress their policies to support mental health promotion and prevent the onset of mental health difficulties. It is intended that the framework can be adapted or tailored by elite sporting organizations based upon their unique cultural, contextual and resourcing circumstances.

## Introduction

Athletes in elite sport are exposed to a range of stressors that have been shown to increase their susceptibility to experiencing mental ill-health, including serious physical injury, poor performance, maladaptive perfectionism and competition for selection (Reardon et al., 2019). This is in addition to general stressors such as adverse life events (Gouttebarge et al., 2019), financial uncertainty, discrimination, or inadequate social support (Purcell et al., 2020; Walton et al., 2021). A recent systematic review suggested that approximately one third of currently competing athletes report experiencing common symptoms of mental ill-health, such as depression and anxiety (Gouttebarge et al., 2019). Retired athletes also report significant mental health concerns (Mannes et al., 2019), often in the context of managing major psychosocial adjustments at the end of their sporting careers, including lifestyle changes, issues regarding their personal and social identity, and financial challenges (Park et al., 2013; Brown J. C. et al., 2017; Sanders and Stevinson, 2017).

Although elite athletes’ susceptibility to mental health concerns appear to be comparable to the general population (Rice et al., 2016), they may be less inclined to seek support for their mental health, citing reasons such as increased stigma and poor mental health literacy (Gulliver et al., 2012; Kaier et al., 2015). Athletes also report fearing potential consequences should they seek help, including loss of selection or even loss of their career (Gulliver et al., 2012; Castaldelli-Maia et al., 2019).

Though there is a rapidly developing evidence-base on mental health in elite athlete populations (Rice et al., 2016; Gouttebarge et al., 2019; Reardon et al., 2019; Küttel and Larsen, 2020; Poucher et al., 2021), mental ill-health can impact other individuals working within high-performance sport (Olusoga et al., 2009). Coaches also experience distinct sport-related stressors such as pressure to succeed, excessive work load, lack of job security, frequent travel, and isolation (Kim et al., 2020; Baldock et al., 2021; Hill et al., 2021), with rates of depression symptoms similar to the general population (Kim et al., 2020).

High-performance environments, by definition, focus heavily on outcomes of success and achievement. This focus can contribute to cultures that do not equally acknowledge and resource athlete, coach and support staff mental health and wellbeing. High-performance environments must be scrutinized for their impacts on individuals, given growing evidence that mentally unhealthy environments can increase the risk for developing mental ill-health more generally (Harvey et al., 2017; Memish et al., 2017). Taking steps toward creating mentally healthy environments for all stakeholders in elite sports organizations will likely have a positive impact on the wellbeing of athletes, coaches and staff, which in turn may positively contribute to athletic performance (Smittick et al., 2019).

## Existing Mental Health Guidelines and Frameworks

Several major sports organizations have published or made publicly available frameworks related to supporting elite athlete mental health, which primarily emphasize the need to build awareness of, and support for, mental wellbeing and/or respond to athletes identified as being “at risk” of a mental health condition (e.g., Australian Football League Players’ Association, 2014a,b; Queensland Academy of Sport, 2014; NCAA Sport Science Institute, 2016; Australian Football League, 2020). The International Olympic Committee (IOC) recently published a comprehensive toolkit to assist Olympic stakeholders (including International Federations and National Olympic and Paralympic Committees) to develop and implement initiatives to protect and promote the mental health of elite athletes (IOC, 2021). Other sporting bodies or associations have published consensus or position statements regarding athlete mental health and wellbeing (Moesch et al., 2018; Schinke et al., 2018; Henriksen et al., 2019, 2020; Reardon et al., 2019; Van Slingerland et al., 2019; Chang et al., 2020), many of which again highlight the need for mental health literacy in athletes as a way to improve help-seeking attitudes and behaviors. A recent systematic review of 13 mental health position statements indicated convergent themes surrounding athlete mental health, athlete support systems, mental health plans, provision of mental health care and managing high risk events (Vella et al., 2021). However, the authors reported that consistency between statements and quality of development (e.g., stakeholder engagement) were low.

Existing guidelines, frameworks and toolkits are vitally important in building the capacity of elite sporting organizations to respond to mental health, but the broader influencing systems or ecological factors are not always considered and rarely addressed in detail. In order for elite sporting organizations to provide optimal mental health support to their athletes, coaches and support staff (e.g., sports medicine practitioners), consideration must be given to individual, contextual *and* systemic needs (Taylor et al., 2012; Rice et al., 2020b).

Current elite sport guidelines identify specific areas that can be addressed to bolster athlete wellbeing, such as the sport’s duty of care to athletes and sport staff, considerations of different stages in an athlete’s career (e.g., junior level to retirement) as well as privacy, confidentiality and ethical issues pertaining to an athlete’s mental health (such as if or how disclosure occurs within the sport as part of a clinical management plan). However, to our knowledge, no framework has utilized peer-reviewed evidence and stakeholder engagement to develop actionable recommendations that sporting organizations can use to promote mental wellbeing in their sporting context, inclusive of environmental or culture factors that can hinder or promote mental wellbeing.

## Creating an Evidence-Informed Framework for Mental Health Promotion in Elite Sport: Foundational Concepts

### An Ecological Systems Approach

**FIGURE 1:**
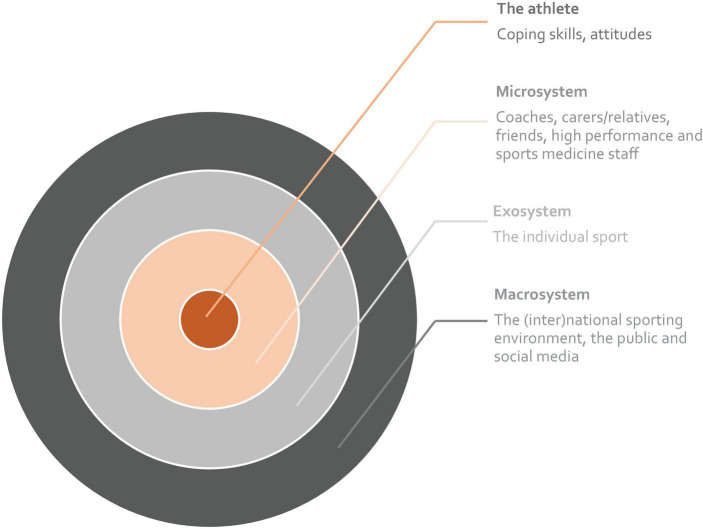
An ecological systems model for responding to elite athlete mental health (from Purcell et al., 2019).

Sporting organizations should aim to address the needs of athletes, coaches and support staff, and the factors that impact upon their mental wellbeing, while simultaneously striving to optimize the environment in which these individuals work, train and compete (Reardon et al., 2019). Recognizing that the mental health of athletes exists within the context of the wider sports system (Coyle et al., 2017), our framework is grounded in an ecological system approach (Bronfenbrenner, 1992) recognizing that the athlete is inseparable from their teammates/colleagues, coaches and support staff, and family or primary supports, as well as their sporting organization. To some extent, this also extends to the relevant national or governing sporting body (see Figure 1). This model, originally described by Purcell et al. (2019) focuses on the transactional relationships between an individual athlete and the broader social and cultural contexts that they inhabit.

### Flourishing, Languishing, and Thriving

**FIGURE 2:**
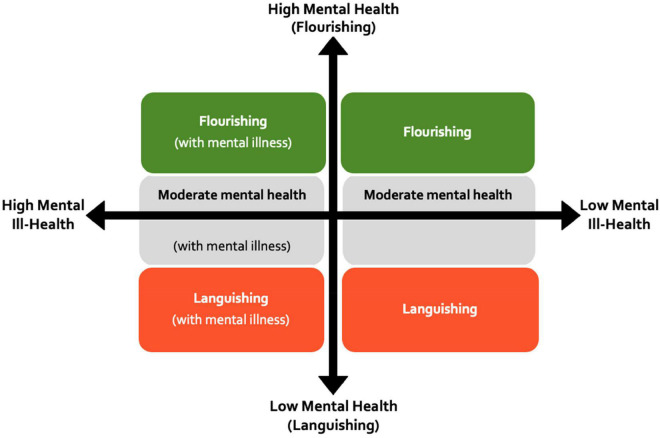
Holistic conceptualization of mental health based on Keyes (2002, 2005).

Our framework is also predicated on the view that mental health and mental illness should be considered as discrete but related constructs (Keyes, 2002, 2005). Through this lens, an athlete, coach or staff member could maintain good mental health (flourishing) even while living with a mental illness, or conversely, experience poor mental health (languishing) in the absence of mental illness (see Figure 2). The terms “flourishing” and “languishing” refer to the presence or absence of a range of factors relating to both wellbeing (positive mood, life satisfaction) and positive functioning (e.g., self-acceptance, personal growth, purpose, autonomy, social connection, among others) (Keyes, 2002, 2005). For example in general clinical populations, “flourishing” has been reported amongst 22% of individuals experiencing substance use disorders (McGaffin et al., 2015) and 28% of those with schizophrenia spectrum disorders (Chan et al., 2018). Therefore it is possible for an athlete with a diagnosed mental disorder to still engage with their sport and experience positive states of wellbeing, such as having positive self-regard, continued personal and professional growth, and meaningful social connections. This framework has been proposed as a useful conceptualization of the full range of mental states that exist within elite sport (Uphill et al., 2016; Lundqvist and Andersson, 2021) and highlights the need for both the promotion of high wellbeing in addition to the prevention of mental ill-health. In elite sport settings, athletes who identify that their sporting environments are less supportive are more likely to be categorized as languishing (Küttel et al., 2021). We posit that the concepts of flourishing and thriving are important for underpinning stigma reduction strategies in elite sport, and ensuring that athletes or staff who are experiencing mental health symptoms remain engaged and connected to the sporting environment, if so desired.

Flourishing, as described above, relates to the presence of hedonic wellbeing (e.g., positive affect) and factors of positive life functioning. The term “thriving” is often used in the sporting context to describe a combination of high wellbeing (i.e., flourishing) and a sustained high level of performance (Brown et al., 2018). Specifically, athlete thriving has been characterized by positive attitudes, motivation, continuous holistic development and a sense of belonging (Brown et al., 2018). From a sample of athletes, coaches and sports staff, Brown et al. (2018) identified a range of contextual and personal factors to promote thriving. Contextual factors included (i) strong interpersonal relationships within the sporting organization, (ii) perceiving support from coaches, teammates, support staff and families, (iii) supportive training environments (e.g., appropriately challenging training), and (iv) experiencing appropriate levels of pressure. Personal factors that athletes, coaches and support staff identified as facilitators of thriving included (i) desire and motivation, (ii) trust and commitment to developmental process, (iii) self-belief, (iv) goal setting, (v) understanding of both personal and sporting demands, (vi) ability to manage stressful situations and (vii) positive mental states (Brown et al., 2018). Several of these factors relate to the ability to promote or maintain self-directed motivation, commitment, understanding and self-regulation, aligning with principles of Self-Determination Theory (Deci and Ryan, 2012).

### Self-Determination Theory

Self Determination Theory (SDT) posits that humans have basic psychological needs for *autonomy, competence and relatedness*, which must be met in order for the individual to experience optimal wellbeing (Deci and Ryan, 2012; Shannon et al., 2020). *Autonomy* refers to a perception of choice and self-directedness, *competence* refers to a feeling that one has ability and opportunity in sport, and *relatedness* is defined as a sense of caring, connectedness and safety with others (Hodge et al., 2008). Mental wellbeing and levels of intrinsic motivation to engage in health-related behaviors may vary in elite athletes according to the extent to which the environment in which they operate satisfies their basic psychological needs (Ryan and Deci, 2000; Deci and Ryan, 2012). SDT provides a useful framework for understanding how elite sport organizations can create an environment to meet these psychological needs (Ryan et al., 2008). Elite sporting environments that are supportive of, and nurture, stakeholders’ needs for autonomy, competence, and relatedness are likely to promote self-development and wellbeing among their athletes (Bartholomew et al., 2011; Mahoney et al., 2014; Küttel and Larsen, 2020; Shannon et al., 2020). In one longitudinal study of a large and diverse sample of athletes assessed at three time-points, a predictive relationship was observed between athletes’ experiences of autonomy, competence, and relatedness, and their subsequent reported levels of thriving (Brown et al., 2021), irrespective of characteristics such as age, gender, or competition level. This work replicates and builds upon Brown and colleagues’ studies illustrating meaningful relationships between basic psychological needs and thriving in sport (Brown D. J. et al., 2017; Brown et al., 2020).

## A Proposed Elite Sport Mental Health Promotion Framework

The current framework originated from a forum attended by 35 athlete wellbeing and sports medicine staff from a range of major Australian elite and professional sports, held in Melbourne in June 2019. Attendees emphasized the various and disparate ways that sports were approaching mental health promotion and lamented the lack of a “road-map,” based on evidence or best practice principles, that could be adapted to improve mental health promotion and cultural change within their own contexts. The need for, and utility of, a road-map or framework for mental health in elite sport was unanimously endorsed by the attendees, and the authors committed to leading the development process, including conducting a literature review and stakeholder engagement on what constitutes best-practice principles in the absence of research evidence. The framework was developed using an iterative process, where gaps in the evidence-base were augmented by stakeholder consensus on principles considered critical to promoting and supporting mental health in elite sporting contexts. The following sections outline the key guiding principles and supporting evidence of the framework, followed by actionable recommendations to promote mental health in elite sport. The guiding principles have been divided into two sections, where the first section discusses promoting mental health and supporting a cultural shift toward prioritizing mental health and wellbeing, while the second section discusses promoting mental health literacy and key protective factors for mental health.

1. PROMOTING MENTAL HEALTH AND WELLBEING (AND SUPPORTING CULTURAL SHIFT)

This section includes evidence and best practice related to the promotion of mental wellbeing, with an emphasis on promoting a *cultural shift* away from a “win at all costs” narrative, to supporting mental health in the high-performance context, and creating psychologically safe and mentally healthy environments that allow athletes, coaches and support staff to thrive.

1.1 Strive to improve the narrative toward mental health in elite sports settings, including conceptualizing mental health and physical health as inseparable

A fundamental principle for sporting organizations to strive for is that athletes, coaches and support staff should be *intrinsically valued*, separate to their sporting role, performance and success. Demonstrating this philosophy throughout decision-making processes will help sports organizations to proactively set the tone regarding their commitments to equally support both physical and mental health (Reardon et al., 2019). Indeed, factors associated with poor mental health (e.g., high stress, intrusive thoughts, impaired cognition) can lead to an increased risk of physical injury (Wiese-Bjornstal, 2010). Akin to physical health, mental health allows individuals to function, cope with stress, perform meaningful work and contribute to society (Gorczynski et al., 2020). As an extension of this philosophy, athlete support and development strategies should incorporate athletic, psychological, psychosocial, academic-vocational and financial components, in line with the Position Stand of the International Society of Sport Psychology (Schinke et al., 2018) (also see 2.2).

Since elite sports value mental toughness (and stoicism, to varying degrees), many individuals may feel reluctant to disclose their experiences of mental ill-health. Given that traditional organizational structures contribute to power imbalances within many sports, organizational processes and communications should be clearly led and supported via a top down approach, with ownership/governance, executive, and coaching staff all supporting a common goal of valuing the mental wellbeing of all individuals. To engage athletes and garner buy-in, any stigmatized narratives that surround mental health must be shifted. Stigma is one of the leading deterrents of help-seeking in elite sport (Gulliver et al., 2012; MacIntyre et al., 2017). Organizational efforts to normalize and validate mental health in elite sports are critical to reducing stigma and improving help-seeking behavior (Moesch et al., 2018). Leveraging openness may foster a non-judgemental environment where staff and athletes are able to engage in open discourse around mental health and wellbeing.

The role of the coach (and role models) in the sporting environment are critical here, as coaches have the potential to help shape team cultures that normalize, destigmatize and are supportive of mental health and help-seeking (Bissett et al., 2020). Coaches who successfully enhanced their teams culture reported doing so by clearly communicating their team’s values (Schroeder, 2010). Coaches should explicitly communicate with athletes that their mental health is valued, promote help-seeking where indicated (including medical help-seeking for physical injuries or concerns) and proactively model this conduct by eliminating language or practices that stigmatize mental illness and mental health help-seeking (e.g., around “toughening up” or similar), including derogatory labeling (Bissett et al., 2020).

These principles support the recommendations (see Table 1 following these sections) to develop and disseminate a Mental Wellbeing Declaration, design personalized development plans, and provide opportunities for a mental health practitioner to work within the sporting environment.

1.2 Create environments that foster wellbeing and strengthen protective factors for mental health

**TABLE 1:** Recommendations.

Recommendations	Framework references
Develop and disseminate through co-design principles*, an individual, team and/or organizational Mental Wellbeing Declaration that outlines how the organization will promote mentally healthy environments and details the wellbeing outcomes for athletes and personnel.	1.1–1.5 Promoting mental health
Establish and define outcome measurement for athlete mental health, and continuously monitor to improve organizational capacity to reach these outcomes.	1.2 foster wellbeing; 2.2 person-centered care
Design personalized athlete development plans to address the physical health and mental wellbeing needs of each individual athlete.	2.2 person-centered care; 1.5 respecting diversity
Ensure a workforce capacity plan is activated to increase mental wellbeing capabilities of sporting organizations. E.g., include minimum accreditation standards and qualifications for mental health practitioners**; ensure competency-based mental health literacy, help-seeking and diversity education is incorporated into minimum compliance education modules.	1.1 improve the narrative; 1.4 ensure basic safety; 1.5 respecting diversity; 2.1 tailoring psycho-education
Create safeguarding policy and procedure for appropriate behavioral conduct, confidential and supportive complaint processes, and dissemination of these policies	1.4 ensure basic safety
Provide opportunities for a mental health practitioner to be embedded within the organization to work with athletes and staff in improving narratives around mental health in sporting environments.	1.1 improve the narrative; 1.2 foster wellbeing; 2.3 opportunities for self-development
Promote healthy and diverse avenues for social support in athletes’ and stakeholders’ sporting and non-sporting lives.	1.3 promote social support
Prepare athletes (and coaches) for key transitions by promoting the development of a non-athletic identity among athletes throughout all stages of their career.	2.4 strengthening external identity; 2.5 support key transitions
Aid development of athletes’ self-management and coping skills to prepare them for sporting (and non-sporting) challenges.	2.3 opportunities for self-development 2.5 support key transitions
Provide bespoke training to coaches surrounding mental health literacy, need-supportive coaching and diversity/cultural awareness, to assist in the promotion of mental wellbeing among their athletes/themselves.	2.1 tailoring psycho-education; 1.5 respecting diversity; 1.2 fostering wellbeing
Provide planned transition programs to support athletes through voluntary or involuntary retirement from sport.	2.2 person-centered care; 2.5 support key transitions
Ensure visibility of diversity to support minority groups by creating on open and inclusive environment that supports unique needs.	1.5 respecting diversity
Develop procedures for the provision of feedback (performance and wellbeing) to and from coaches, athletes and staff.	1.2 fostering wellbeing; 2.2 person-centered care

*Co-design refers to the process of bringing people with different perspectives, needs, knowledge and skills to collaboratively develop a response to a concern (Zamenopoulos and Alexiou, 2018).

**A mental health practitioner refers to a qualified mental health professional such as a psychiatrist, psychologist, mental health nurse, occupational therapist or social worker.

Supportive environments should be underpinned by a culture of “psychological safety” among stakeholders within the organization. Creating an environment that is perceived as being “psychologically safe” reflects a climate of interpersonal trust, mutual respect, acceptance and civility (Edmondson, 1999), where individuals can take interpersonal risks, such as ask questions or seek feedback without fear of negative repercussions, such as humiliation, rejection or criticism (Edmondson, 2004). Psychologically safe environments enable individuals to “be themselves,” without the need to “mask” attributes, values or beliefs. While research is yet to investigate the role of psychological safety in promoting mental wellbeing in elite sport, emerging studies have reported a positive association between psychological safety and team performance (Smittick et al., 2019) and mediating effects of psychological safety between team identification and wellbeing through reduced burnout (Fransen et al., 2020). An abundance of research in non-sporting work contexts has reinforced the positive outcomes of psychological safety (Newman et al., 2017; Albritton et al., 2019). Creating climates that are perceived as psychologically safe facilitates active participation among stakeholders (Edmondson, 1999).

According to SDT, sporting organizations should aim to create and maintain an environment that supports autonomy, competence and relatedness (or connection/connectedness) amongst all individuals. Allowing individuals to take a greater position of autonomy will improve intrinsic motivation to engage in health-related behavior. An environment that supports autonomy provides athletes, coaches, and support staff with the rationale for tasks, opportunities for input and decision making, and acknowledges their perspectives and feelings (Mageau and Vallerand, 2003). Coaches are well-placed to provide athletes the opportunity for greater self-direction, which can lead to higher intrinsic motivation (Amorose and Anderson-Butcher, 2007; Gillet et al., 2010; Banack et al., 2011) and promote athlete wellbeing and prevent burnout (Langan et al., 2015). The interpersonal style employed by coaches is highly influential (Vallerand and Losier, 1999). Emotionally abusive behaviors, such as shouting, belittling, and using degrading comments and humiliation pose a threat to the mental wellbeing of athletes (Gervis and Dunn, 2004; Kavanagh et al., 2017). That this can include burnout and maladaptive coping calls into question the ethics of such approaches (Bartholomew et al., 2011; Balaguer et al., 2012; Stirling and Kerr, 2013). Conversely, interventions that promote positive coaching communication styles have led to improved athlete mental health (Smith et al., 1995, 2007) and maintain psychologically safe environments.

A sporting environment that supports competence should provide adequate challenges and feedback to athletes to bolster feelings of self-belief and accomplishment (Brown et al., 2018). Pressure on the athlete to perform, as well as pressure placed on coaches (and support staff) for success (from funders, governing bodies or boards) can hinder thriving and influence coaches into enforcing a controlling style that is not needs-supportive (Berntsen et al., 2019) and does not foster autonomy. Building autonomy and competence requires the use of appropriate feedback to offset the negative impacts of perceived pressure and maintain motivation (Cheon et al., 2015). Specifically, feedback that is factual, non-judgemental, concrete, formative, changeable and conveys high yet realistic expectations, is beneficial for building intrinsic motivation and creating competence support (Mageau and Vallerand, 2003). The use of these techniques should be applied when providing feedback to any member within the sporting environment (i.e., not just for athletes).

Lastly, leveraging social connection will promote feelings of relatedness in the elite sporting environment. Social support is among the most influential factors enhancing mental wellbeing in athletes (Küttel and Larsen, 2020; Purcell et al., 2020; Walton et al., 2021), while poor social connection is associated with low self-esteem, depressive symptoms and psychological distress (Baumeister and Leary, 1995; Gouttebarge et al., 2015). Within sports organizations, high quality, supportive relationships have been associated with several positive outcomes, including improved psychological health, adaption to stress and improved performance (Burns et al., 2019). Interestingly, Walton et al. (2021) demonstrated that athletes generally prioritize personal relationships outside sport for support, rather than turning to those within their sporting environment. While 7% of the elite athlete sample endorsed their sport psychologist as primary source of support, less than 2% selected their coach. Instead, friends, family, and partners were much more readily relied on as main sources of support for athletes. This may support the view that athletes do not feel sufficiently safe to disclose mental health concerns within sporting environments, and instead turn to trusted and reliable personal relationships.

The principles of SDT and psychological safety uphold the recommendations (Table 1) to develop procedures for the provision of feedback, providing bespoke training for coaches (and/or other stakeholders where relevant) to create environments that are supportive of basic human needs (e.g., autonomy, competence, relatedness), and promote healthy and diverse avenues for social support. Combined, these elements facilitate mentally healthy environments by promoting socially safe interpersonal relationships and communication styles.

1.3 Promote healthy social support in stakeholders’ sporting and non-sporting lives

There are various avenues for promoting relatedness, including peer support (from the team or fellow athletes), supervisor support (coaching/support staff), formal support (mental health clinicians), and external support (family, friends, partners). Coaching staff should strive to engage in genuine, personalized conversations with athletes where possible, since athletes feel more supported by coaches who relate to them in an empathetic way (e.g., asking about their lives and interests outside sport) than coaches who merely communicate technical knowledge (Burns et al., 2019). Developing relationships (coach-athlete and athlete-athlete relationships) in this way may also make an athlete feel more valued as a person, rather than only feeling valued for their skills and performance.

To strengthen external support networks, sporting organizations should aim to provide opportunities for athletes, coaches and support staff to integrate their family or peer network within the sport where possible. For example, social functions and events organized by sporting organizations should ideally include athletes’ family or peer support network where possible, and sports organizations should facilitate the opportunity for family members to accompany athletes and coaches during extended periods of travel away from home where possible.

1.4 Ensure the basic safety of athletes and others in the sports setting, including protection from any risks to physical safety and creating cultures with zero tolerance toward known contributors to mental ill-health (including abuse, racism, and discrimination)

Abuse or maltreatment occurs in elite sporting environments, and contributes to impaired mental health. This includes acts of neglect and/or physical, sexual, and emotional abuse, and varying forms of bullying, harassment, exploitation, institutional maltreatment, and abuse or assault (Stirling, 2009). Central to the potential for athlete maltreatment (especially junior athletes) is the inherent power imbalance that exists between the athletes and those responsible for decisions that affect their careers, including selection, training priority and medical treatment. Coaches and support staff (including sports medicine practitioners) all hold positions of power and should be explicitly aware of the effect that this can have on athlete wellbeing.

Safeguarding strategies are crucial to ensure that athletes are not subject to such negative experiences (Mountjoy et al., 2015), and include developing safeguarding policies and clear procedures for responding to safeguarding concerns, communicating to athletes where they can seek advice and support, eliminating or minimizing risks to athletes, developing codes of conduct for behavior in the sport setting, ensuring appropriate recruitment and training processes and ongoing monitoring and evaluating of safeguarding compliance and effectiveness (International Safeguarding Children in Sport Working Group, 2016).

All individuals working within elite sporting organizations should be given information regarding complaints processes, which should be easily accessible to athletes of all ages and levels of competition. This should involve anonymous (“whistle-blower”) avenues as well as confidential complaints. Policies and procedures should be developed and communicated about protecting complainants from negative consequences to their careers.

These basic safety principles designate the recommendations to create safeguarding policy and procedure for appropriate behavioral conduct, confidential and supportive complaint processes, and dissemination of these practices to all stakeholders.

1.5 Develop respect for diversity and individual differences within sport

Diverse and minority groups are likely to face a range of both sport-specific and culture-specific stressors that may impact their mental health (Ryba, 2017). For example, First Nations athletes have recounted the challenges associated with relocation to unfamiliar cultural communities (Schinke et al., 2006), while a lack of cultural understanding by coaches, teammates and staff can lead to alienation, marginalization, self-imposed retirement and depressive symptoms (Blodgett and Schinke, 2015; Schinke et al., 2018). Cultural factors can also act as barriers to help-seeking for mental health concerns, such as gender role beliefs (e.g., high masculinity and toughness in male athletes), religious beliefs (e.g., low acceptability of mental health concepts) and dependence on economic benefits from sport participation (Castaldelli-Maia et al., 2019).

Sports organizations should strive to create environments that are inclusive and supportive of diversity to reflect the varying needs of athletes. Mentally healthy sports environments are respectful and inclusive of cultural, identity (including sexuality and gender), religious and linguistic needs of individuals, whilst also recognizing how these factors intersect. Culture-specific support for athletes should be implemented where appropriate, such as access to culturally competent mental health providers, cultural spaces (e.g., a prayer room) and observance of cultural practices (e.g., kinship connections). While developing individual development plans for each athlete, cultural and identity differences should be considered. This requires an individualized approach to athletes that takes into account their own particular contextual needs.

At the organizational level, creating a capable workforce will enable identification of those contributing to, or experiencing, marginalization and discrimination. This allows for early identification and intervention to reduce or prevent mental wellbeing concerns. Further, “visibility” (seeing yourself in the environment) can be created via employment of staff from different cultures, races, genders, religions, and sexualities. Celebration of diversity directly opposes “denial of visibility,” that pressures individuals to conform to the accepted or visible archetype (e.g., heteronormativity, hegemonic masculinity) by denying or prejudicing minority groups (Amodeo et al., 2020). This pressure can lead to distress, depressive symptoms, withdrawal, and shame (Symons et al., 2017). While there has been some recent progress in elite sports organizations in terms of recognizing the importance of diversity, sports should strive to embrace diversity in their organizations wherever possible.

The recommendations that accompany these principles include implementing bespoke training for diversity/cultural awareness, ensuring competency-based education is incorporated into minimum compliance modules and ensuring visibility of diversity to support minority groups by creating open and inclusive environments.

2. PROMOTING MENTAL HEALTH LITERACY AND PROTECTIVE FACTORS

This section focuses on promoting increased understanding about mental health, including enhancing knowledge about risk factors for mental ill-health and enhancing protective factors for mental health, which in turn can help to prevent the onset of mental ill-health.

2.1 Provide sports-specific mental health training that is tailored to the sporting context

Sporting organizations that strive to build mental health literacy are best placed to reduce stigma and enhance help-seeking (Gulliver et al., 2012). Poor mental health literacy is recognized as a notable barrier to help-seeking behavior in sport (Gulliver et al., 2012; Castaldelli-Maia et al., 2019). This can manifest as lack of ability to recognize symptoms of mental ill-health, lack of knowledge about available treatment options, and fear of stigma or negative consequences arising from disclosing a mental health concern. Participation in psychoeducation about mental health should be a minimum compliance requirement that will convey the organization’s commitment to creating a cohesive understanding of mental wellbeing within the sporting environment.

Gorczynski et al. (2020) emphasized that mental health literacy interventions should be tailored to developmental, cultural, social and systemic considerations in elite sporting organizations (for example, factoring in age, type of sport and whether an individual or team sport). Athletes are likely to differ in their expression of poor mental wellbeing compared to the general population, and this needs to be incorporated into psychoeducation delivered by a skilled (e.g., experienced in elite sport) mental health clinician; for instance, differentiating burnout or overtraining from depression (Kreher and Schwartz, 2012).

Acknowledging the broader context in partnership with specific education regarding the sporting organization’s current procedures, avenues to support and commitment to mental wellbeing will provide athletes, coaches and staff with the confidence and know-how to look out for the wellbeing of themselves and others.

This evidence base endorses the recommendations to provide bespoke (i.e., sports-specific) training for mental health literacy and help-seeking and to ensure training is part of minimum compliance modules.

2.2 Promote mental health and wellbeing strategies within sporting organizations that are person-centered and reflect an individual’s changing needs

Sporting organizations that understand and acknowledge that each athlete is unique in their wellbeing support needs will avoid the trap of a “one-size-fits all” mentality or approach. In this regard, opportunities for individual development, support and care should be provided in a *person-centered* manner. Though definitions vary across disciplines (e.g., medicine, disability support), person-centered care relates to honoring the individual, engaging them in participation and facilitating their strengths (Waters and Buchanan, 2017). Person-centered approaches acknowledge that the individual is the expert on themselves and places decision-making in their hands where appropriate. In practice, each athlete, coach and staff member with mental health needs should have a personalized wellbeing care plan developed, where the individual has autonomy to communicate their unique risk and protective factors. Mental health outcomes that are monitored as part of a care plan will vary across individuals, which further helps to avoid a template or “tick-box” style care plan. Equally, an athlete’s needs will change over time, and this should be reflected by updating their wellbeing care plan. Developing the athlete as an autonomous individual, and meeting this innate need, is a key value of the athlete talent development process in elite sports organizations (Henriksen et al., 2010). Furthermore, intrinsic (i.e., self-produced) goals nominated by athletes themselves are associated with greater health, wellbeing and performance (Vansteenkiste et al., 2004; Ryan et al., 2008).

Understanding when an athlete may be at risk of experiencing heightened distress requires both recognition of their unique risk factors *as well as* common contexts for challenges (e.g., serious injury, traveling overseas for competition, or training without adequate social support). In this sense, tailoring care to the individual’s context also plays a role in *preventing* poor wellbeing or mental ill-health. For example, transitioning out of elite sport can often involve a loss of identity and purpose. Adequate self-management skills are crucial during periods of career transition (Bernes et al., 2009). Early intervention programs aimed at broadening the life skills of elite athletes may partially mitigate the risk associated with retirement (Anderson, 2012).

These principles and supporting literature underpin the recommendations to create personalized development plans and establish and define outcome measurements for mental health.

2.3 Provide ample opportunity for athletes, coaches and staff to develop effective self-management skills, with support from a mental health clinician

Sporting organizations, via engagement with mental health clinicians, medical teams or other support staff, can use appropriately shared information from tailored plans to recognize times when further support may be needed and help facilitate the development of athlete and coach self-management skills. Self-management skills such as managing stress and emotional regulation, optimal communication styles, or problem-solving techniques, are often under-developed in elite athletes as a result of the performance focused environments in which they typically operate (Anderson, 2012). Sporting organizations should assist athletes and coaches to develop a range of self-management and adaptive coping skills (such as cognitive or behavioral coping strategies, as opposed to avoidance as a coping style) particularly for use during periods of psychological distress (Purcell et al., 2019). Developing athlete use of adaptive coping strategies to overcome problems has a significant impact on the development of a resilient profile in elite athletes (Belem et al., 2014). The ways by which athletes appraise and cope with personal and athlete-specific stressors can be a powerful determinant of the impact the stressors have on both their mental health and their sporting success (Rice et al., 2016). The ability to manage stressful situations is a key indicator of thriving in elite sport (Brown et al., 2018).

These principles and supporting literature underpin the recommendations to aid development of self-management and coping skills.

2.4 Promote the development of a non-athletic identity among athletes and coaches throughout their career

Athletic identity represents the extent to which an athlete’s conception of their self-identifies with their role as a sportsperson (Brewer et al., 1993). Self-concept can encapsulate a range of factors such as perceived values and social networks. Though certain aspects of an athletic identity may aid success (e.g., perfectionism), the same traits are often associated with poorer mental wellbeing (Chang et al., 2020). Furthermore, individuals with a unidimensional athletic identity are more susceptible to negative outcomes upon retirement, including psychological distress, symptoms of depression and poor vocational/employment and financial adjustment (Knights et al., 2016; Sanders and Stevinson, 2017).

Conversely, non-athletic factors have been associated with the quality of sport career transitions and adjustment to life after retirement from sport (Erpič et al., 2004). An athlete who has other leisure, education or career pursuits in which to participate and find meaning or purpose, will likely find career transitions to be easier (Anderson, 2012; Bennie et al., 2021). However, developing skills or acquiring qualifications often takes time and athlete retirement is frequently unplanned or involuntary. Considering this, the development of a *non-athletic* identity in preparation for the eventual end of competitive sport must occur at all phases of the sporting career, from junior levels, to elite on-boarding and throughout the career (Park et al., 2013). Additionally, sports organizations should assist athletes who are retiring from their sport with development of a comprehensive pre-retirement plan (Chang et al., 2020). Sports organizations should also assist coaching staff to develop a non-sporting identity, which may also provide a positive model of this behavior to athletes.

This evidence base supports the recommendation to promote non-athletic identity throughout all stages of athletes’ (and coaches’) careers.

2.5 Equip athletes and coaches with necessary skills and support them during key sport-related transitions, including during the transition into and out of elite sporting environments

A range of key “transitions” have been identified in elite sport that can lead to problems with mental wellbeing, including transitioning *into* elite sport (Bruner et al., 2008; Stambulova et al., 2020), following major injury (Putukian, 2016), trans-national or “cultural” migration (e.g., to a new team or organization) (Ryba et al., 2018) following major events (e.g., returning from the Olympics/Paralympics or other major events) (Howells and Lucassen, 2018; Bennie et al., 2021), as well as the transition out of sport into retirement. Each of these contexts requires careful consideration of both preventative and responsive measures.

Athletes should be prepared for, and supported through, a range of key sport-related transitions.

Stambulova et al. (2020) highlighted that understanding of each of these experiences – or their combination—requires a holistic lens, incorporating athletic, psychological, psychosocial, academic/vocational or financial factors or perspectives. Sporting organizations must endeavor to provide adequate career-long psychological support services for athletes and coaches, which acknowledges the many inter-related transitional stages likely to be experienced. Sporting organizations should ensure that athletes and others within elite sport environments are aware of the stressors they may encounter related to major career transitions and ensure support is available via appropriate referral pathways to mental health clinicians when appropriate (see for example, the NFL rookie career transition program and the AIS Mental Health Referral Network) (Rice et al., 2020a; NFL, 2021). This involves discussing the need to maintain mental and physical health and the resources available to players in order to support their wellbeing needs.

These principles and supporting literature underpin the recommendations to provide planned programs/support for key transitions.

## Actionable Recommendations to Support Framework Implementation

Table 1 provides actionable recommendations that sporting organizations can adopt and tailor to meet their unique needs, that correspond to the sections within the framework.

The recommendations provided are specific yet flexible, in order to allow for relevant tailoring. For instance, we recommend that sporting organizations promote development of a non-athletic identity, yet we do not prescribe specific programs. Sporting organizations may opt to embed opportunities for further education, programs to develop vocational skills, or set aside time for athletes’ personal interests/past-times. The organization’s resources (e.g., funds, people, schedule availability, accessibility to facilities/experts, etc.) will also influence how these recommendations may be best implemented. The overarching principles that should be considered when translating these recommendations into practice is to collaborate with the key stakeholders, seek feedback, and continuously evaluate. Just as this framework was developed in collaboration with sporting staff, expert researchers and mental health practitioners, is it crucial that sporting organizations, coaches, athletes and sports staff be consulted and actively involved in the implementation process. When initiatives are in place, those involved should be provided avenues for feedback regarding what is and is not working. This process is crucial as it may highlight discrepancies and suggest areas that need further development. For instance, Moore (2016) found that athletic directors perceived mental health resources and psychosocial services for student athletes to be more readily available than the athletes’ perceptions. Continual evaluation of how initiatives are perceived and how they may be influencing targeted outcomes is imperative to creating a thriving environment where stakeholders flourish.

## Discussion

This paper presents a collaboratively developed and evidence informed framework to guide best-practice in mental health and wellbeing promotion within sports settings. In high-pressure success-oriented environments, stakeholders can become caught up in the desire to win, losing sight of the wellbeing needs of themselves and others in order to achieve (Berntsen et al., 2019). Sporting organizations that proactively develop and implement strong policies and practices will not only create psychologically safe, thriving environments for their athletes, coaches and staff, they will lead the way for others to follow suit.

Recommendations listed above are underpinned by the extant evidence from elite and professional sporting contexts, although more research is needed to guide mental health supports in elite sport. For instance, there may be significant differences between team and individual sport environments. Preliminary research comparing individual and team sport athlete stressors and coping emphasize specific team-related stressors such as selection pressure and letting team-mates down (Nicholls et al., 2007), and a tendency for individual sport athletes to favor emotion-focussed coping in response to stressors, compared to the use of communication techniques in team-sport athletes (Nicholls et al., 2007). Our framework designates teammates as key elements of the sport “ecology,” but this may not be as relevant for individual sport settings. Further research into the roles of connectedness, social support and cohesion in team and individual sport settings will enhance the way the framework may be tailored to each context.

The framework would also be advanced by the formulation of a unified definition of, and ability to accurately measure, athlete wellbeing (Giles et al., 2020). Within current sport psychology research, athlete wellbeing is an indeterminate and inconsistently defined construct, leading to an array of operationalized variables within the literature (Lundqvist, 2011). Frequently used measures of athlete wellbeing include life satisfaction, positive affect, self-esteem and subjective vitality (Giles et al., 2020). Though such concepts are inevitably linked with aspects of wellbeing, individually they may not encapsulate the entirety of the construct. Without a recognized and broadly accepted definition, ambiguity and incongruences may compromise understanding, subsequently impacting research and further policy development. Greater clarity of definitions would bolster recommendations within this framework (e.g., establishing and defining outcome measurement for athlete wellbeing, personal development and thriving) and their implementation.

Through the development of this framework, we call on elite and professional sporting organizations to augment policies created for *performance* agendas with policy that is guided by supporting and improving wellbeing (Diener and Seligman, 2004). Recognizing that athletes are “people first” is necessary to improving psychological safety within elite sport. Involving the people for whom the policies are created (e.g., athletes, coaches, high performance staff) in the co-design process will ensure that sporting organization’s policies are best placed to suit their specific needs.

## Author Contributions

RP, SR, SC, and DR contributed to the concept for the framework and manuscript. All other authors contributed to the drafting of the manuscript and take responsibility for the work.

## Conflict of Interest

DR was employed by the company Focus Coaching. The remaining authors declare that the research was conducted in the absence of any commercial or financial relationships that could be construed as a potential conflict of interest.

## Publisher’s Note

All claims expressed in this article are solely those of the authors and do not necessarily represent those of their affiliated organizations, or those of the publisher, the editors and the reviewers. Any product that may be evaluated in this article, or claim that may be made by its manufacturer, is not guaranteed or endorsed by the publisher.
